# Gravity beyond starch: molecular evidence for a parallel gravity sensing mechanism

**DOI:** 10.1093/plphys/kiag087

**Published:** 2026-02-26

**Authors:** Rose McNelly, Thomas Depaepe

**Affiliations:** Assistant Features Editor, Plant Physiology, American Society of Plant Biologists; John Innes Centre, Norwich Research Park, Norwich NR4 7UH, United Kingdom; Assistant Features Editor, Plant Physiology, American Society of Plant Biologists; PlantSynergy, Dpt. Evolutionary Biology, Ecology and Environmental Sciences (BEECA), Faculty of Biology, Universitat de Barcelona (UB), Barcelona 08028, Spain

Unlike most environmental cues on earth, gravity is constant, omnipresent, and unidirectional. As such, it provides reliable positional information that organisms use to orient themselves, regulate their growth, and acquire essential resources (Takahashi et al. 2021). In plants, gravity directs growth and shapes architecture through gravitropism, the directional growth response that orients shoots upward and roots downward. However, this response is fine-tuned by other environmental conditions including light, nutrients, and water (Nakayama et al. 2012; Vandenbrink et al. 2019).

Gravity perception in plants has been explained by the starch-statolith model, in which starch-filled amyloplasts sediment within specialized cells in root caps or the shoot endodermis, triggering auxin transport and redistribution, and differential growth (Darwin and Darwin 1880; Takahashi et al. 2021). This model is supported by extensive experimental evidence, yet aspects of the gravitropic response remained unexplained. Studies of starchless *phosphoglucomutase* (*pgm*) mutants demonstrated that statolith sedimentation is crucial for robust gravity sensing, but these mutants are not completely agravitropic (Wolverton et al. 2011). *pgm* roots retain a persistent, though reduced, gravitropic response, even in the absence of detectable auxin redistribution (Wolverton et al. 2011; Roychoudhry et al. 2025). This raised the possibility that plants possess an additional gravity-sensing mechanism that operates independently of starch-filled statoliths.

In a recent article in *Plant Physiology*, Canaday et al. (2026) investigate unresolved aspects of gravitropism in *Arabidopsis*. The authors exposed *Arabidopsis* seedlings to fractions of Earth's gravitational force (1 g) on the International Space Station (ISS) to modulate the strength of the gravity stimulus. Using these carefully designed fractional gravity treatments in combination with transcriptomic analyses, they provide evidence for a starch-independent gravity-sensing mechanism. In their first assay, root tip angles of wild-type and *pgm* plants were measured under Earth's gravity in response to a phototropic stimulus. As expected, both genotypes exhibited phototropic bending in unidirectional light. In addition, gravity strongly attenuated the phototropic response in wild-type plants and to a lesser extent in the *pgm* mutant, consistent with previous reports (Vitha et al. 2000). The experiment was then repeated aboard the ISS using the European Modular Cultivation System under microgravity conditions. Under microgravity, no significant difference in root angle was observed between wild-type and *pgm* plants. These results indicate that *pgm* does not differ from wild type in its intrinsic phototropic response and supports the conclusion that the altered root angles observed on Earth result from a bona fide starch-independent gravity-sensing mechanism.

To characterize the sensitivity of both starch-dependent and -independent gravity-sensing mechanisms, wild-type and *pgm* seedlings were grown on the ISS in darkness under simulated gravitational vectors ranging from 0.003 to 1 g and changes in root angle and growth observed. At 0.003 g more than 50% of wild-type plants responded, while fewer than 25% of *pgm* plants responded to this gravitational force. Instead, at 0.011 g, 100% of wild-type plants responded, while forces of 0.09 g and 0.38 g were required to elicit responses in 50% and 100% of *pgm* plants, respectively. When combined with the fact that *pgm* statoliths do not sediment (Roychoudhry et al. 2025), these results suggest the existence of a starch-independent gravity response mechanism in *pgm* plants. In addition, these results indicate that distinct forces are necessary for activating each sensing mechanism.

To identify the molecular mechanisms underpinning gravity sensing, the authors investigated the transcriptional response of wild-type and *pgm* plants at 12 simulated gravity vectors from 0.004 to 1 g. They used an RNA sequencing approach and focused on root tips as they had the strongest response. There were large transcriptional changes between wild-type and *pgm* plants at 0.38 g, the gravitational force that induced starch-independent gravitropism in *pgm* mutants. Differentially expressed genes in the wild type were associated with transcription factors and DNA binding; meanwhile in *pgm* mutants, the differentially expressed genes were involved in cell wall remodeling and cell-to-cell communication, the latter being suggested as a putative important mechanism in statolith-independent gravity sensing.

To explore the role of cell-to-cell communication as part of starch-independent gravity-sensing mechanism, the authors selected 6 genes that were differentially expressed at 0.38 g in the *pgm* mutant. The genes encode lipid transferases, a membrane steroid binding protein (MSBP1), and 2 peptide transporters of the USUALLY MULTIPLE ACIDS MOVE IN AND OUT TRANSPORTER (UMAMIT) family. T-DNA insertion mutants of the genes were then phenotypically analyzed. Five of the 6 mutants had altered root angles under an Earth-based gravistimulation experiment. Interestingly, the mRNA of *MSBP1* may be mobile (Thieme et al. 2015), raising the possibility that this gravity-sensing and response mechanism be multicellular. The sixth gene, encoding an extracellular peroxidase, had altered root angles in continuous gravitropic assays, suggesting that extracellular signals might trigger the gravitropic response. Together, these results suggest that cell-to-cell communication is important in the starch-independent gravitropic response, yet the precise molecular mechanism is still unclear.

In summary, the work by Canaday and colleagues represents the most sensitive analysis to date of gravity-sensing capabilities in plants. Their findings offer evidence for a starch-independent gravity-sensing mechanism, which was previously hypothesized (Caspar and Pickard 1989) in parallel to the statolith-dependent gravity perception (Fig. 1). This study identified the threshold (0.38 g) at which the starch-independent mechanism is triggered in starchless mutants. Although the precise signals that initiate the new gravity-sensing pathway remain unknown, the authors suggest that cell-to-cell communication may play a role. In this context, it may be worthwhile to revisit earlier studies proposing that mechanical forces acting on cellular structures, such as the plasma membrane, cell wall, or even organelles, contribute to gravity perception (Lopez et al. 2014). These findings also raise important questions. For example, what signal triggers the starch-independent gravity response? Are the two different gravity-sensing and -responding mechanisms independent, or do they cooperate? Does this novel mechanism operate in noncolumella cells, and might it also operate in shoots? And under what conditions on Earth might this starch-independent pathway be important?

**Figure 1:**
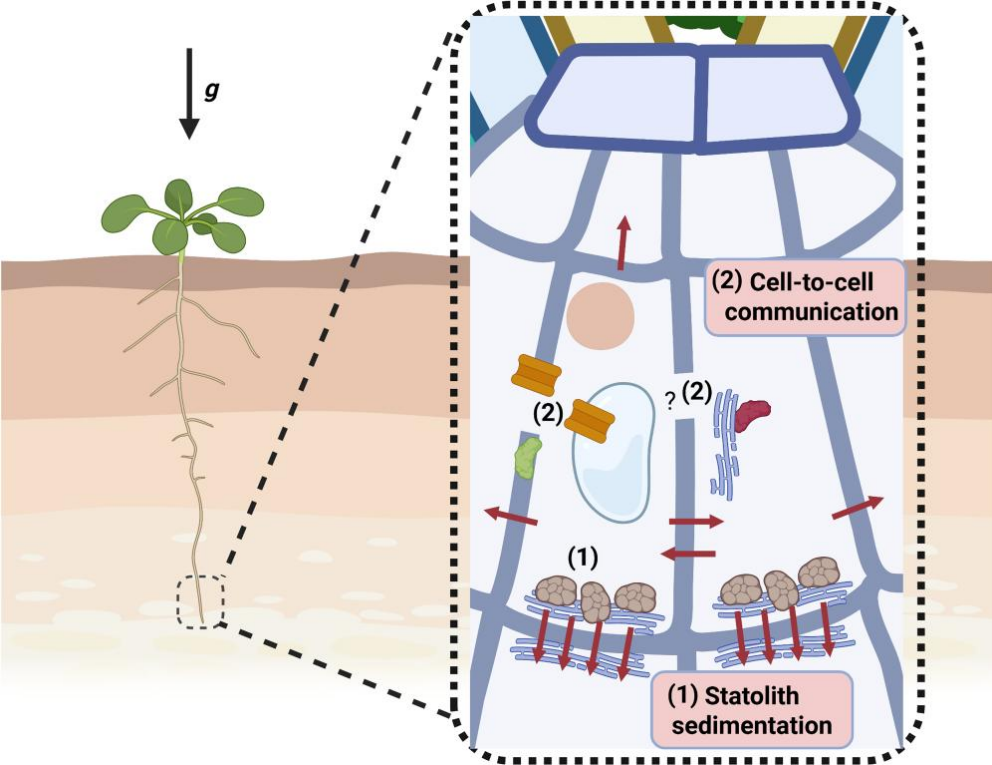
A dual-mechanism for gravity sensing in plants in root tips. Plants perceive gravity vectors (g) and exhibit directional growth toward it. There are 2 mechanisms for gravity sensing: (1) the classical starch-dependent gravity-sensing mechanism, and (2) the starch-independent cell-to-cell communication mechanism (characterized in the Canaday et al. (2026) study). The first mechanism (1) relies on the sedimentation of starch-containing statoliths (brown circular shapes) in the root caps and controls auxin transport (arrows) and growth toward the gravity vector. The starch-independent gravity-sensing mechanism (2) identified by Canaday and colleagues operates independently of statoliths. It is likely to rely on cell-to-cell communication, and the authors postulate the roles of mRNA movement, peptide transporters, and extracellular peroxidases in this process, although the precise mechanism is still unclear (represented by "?"). The figure was prepared in BioRender.

## Recent related articles in *Plant Physiology*

- Luan et al. (2025) revealed how bamboo senses and responds to gravitational and mechanical stimuli across cellular and tissue scales, allowing it to maintain stability during rapid elongation.
- Akita and Miyazawa (2025) investigated how cortical cells and the gene *MIZU-KUSSEI1* coordinate root hydrotropism in Arabidopsis, exploring the cellular mechanisms that guide roots toward moisture gradients.
- Kohler et al. (2024) described how the WEEP protein enhances gravitropism roots and causes negative gravitropism in branches of the peach tree by influencing polar auxin transport.

## Data Availability

No new data were generated or analysed in support of this research.
